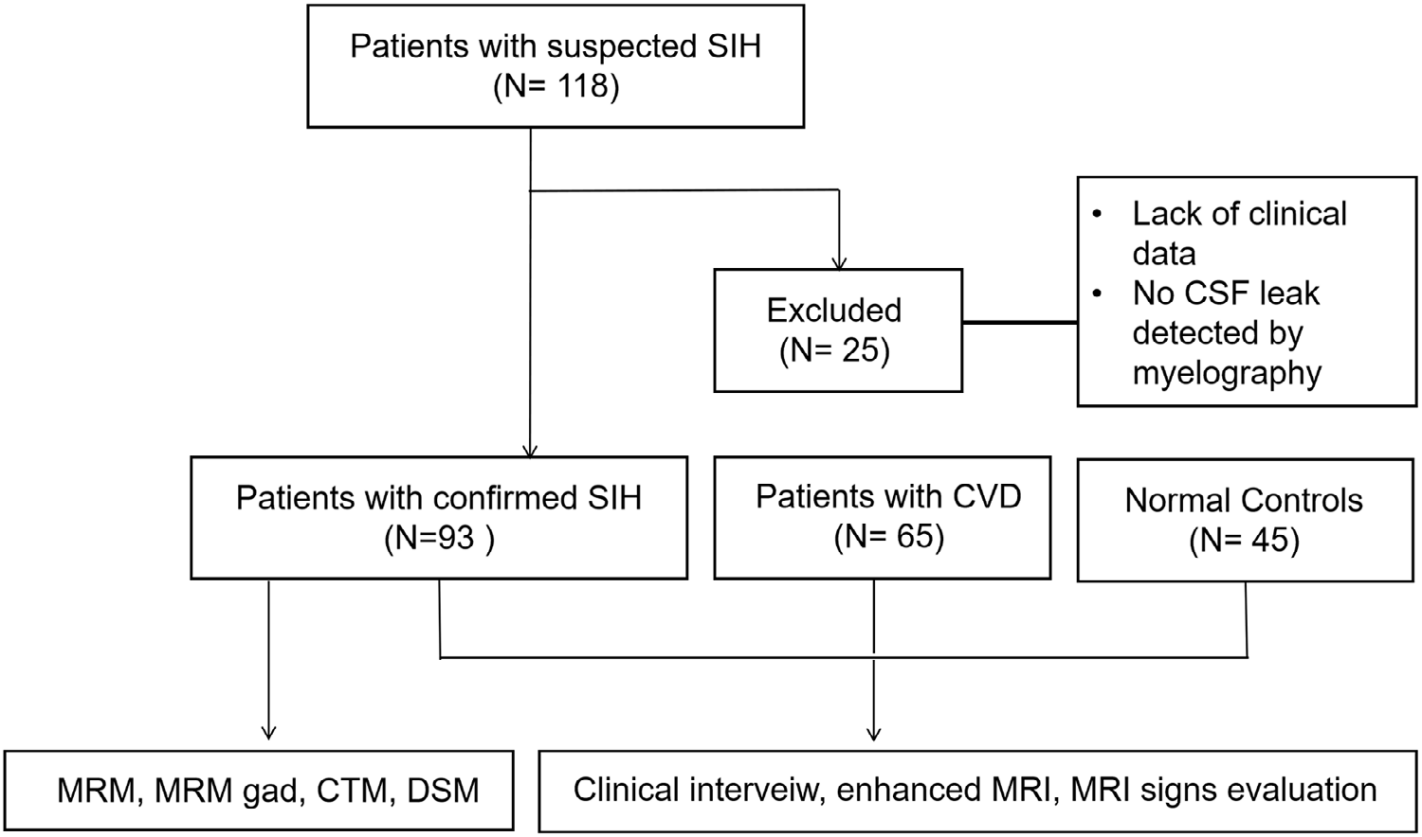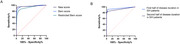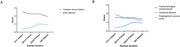# Optimization and Validation of a New Score in Diagnosing Spontaneous Intracranial Hypotension

**DOI:** 10.1002/alz70856_106368

**Published:** 2026-01-08

**Authors:** Kexin Xie, Zhen Wang, Qiang Guo, Tianxinyu Xia, Fei Wang, Hong Ye, Chong Shen, Yan Li, Jiabin Liu, Yanfang Dai, Yanhong An, Zheng Wang, Tengda Liu, Jie Wu, Zhigang Qi, Liyong Wu, Junjie Li

**Affiliations:** ^1^ Xuanwu Hospital, Capital Medical University, Beijing, China; ^2^ Xuanwu Hospital, Capital Medical University, Beijing, Beijing, China; ^3^ Beijing Fengtai Rehabilitation Hospital, Beijing, Beijing, China; ^4^ Beijing Xuanwu hospital, Beijing, China

## Abstract

**Background:**

Diagnosis of spontaneous intracranial hypotension (SIH) relies on detecting cerebrospinal fluid (CSF) leak by myelography and opening pressure by lumbar puncture. However, these intrusive examinations limit the early diagnosis of quick identification. We developed a new scoring system using magnetic resonance imaging (MRI) based on the Bern score to assist in diagnosing SIH.

**Method:**

This case‐control study in consecutive patients investigated for SIH was conducted at a single hospital department from November 2018 to October 2022. SIH patients were diagnosed by ICHD‐3 and with CSF leak detected by myelography. Cerebral venous disease (CVD) patients diagnosed by international criteria and healthy controls were also included for differentiation. Three blinded readers reviewed the brain MRI scans of SIH patients, CVD patients, and healthy controls. Six signs from the Bern score were evaluated in all three groups. The new scoring system was developed from the items of the Bern score using binary logistic regression analysis. Its likelihood of the diagnosis was compared to that of the Bern score and the restricted Bern score.

**Result:**

A total of 93 SIH patients (48 female [51.6%]; mean [SD] age, 41.4 [10.0] years), 65 CVD patients (36 female [55.3%]; mean [SD] age, 44.5 [13.2] years) and 45 healthy controls were studied. Three qualitative signs were selected from the six original signs in the Bern score. Pachymeningeal enhancement and subdural fluid were weighted major (2 points each), and venous sinus distention was weighted minor (1 point). The cut‐off value was 0.5 on a scale of 5 points. The new score showed better discriminatory value compared to the Bern score and restricted Bern score by the McNemar test (*p* <0.001), sensitivity (93.5%), specificity(96.4%), and AUC(0.97). It also showed consistent diagnostic value as disease duration varied.

**Conclusion:**

The new MRI‐based scoring system, derived from the Bern score, effectively predicts SIH diagnosis using qualitative signs, making it more practical for clinical use and facilitating earlier identification and diagnosis of SIH.